# Asymptomatic Cases, the Hidden Challenge in Predicting COVID-19 Caseload Increases

**DOI:** 10.3390/idr13020033

**Published:** 2021-04-09

**Authors:** Brett Snider, Bhumi Patel, Edward McBean

**Affiliations:** School of Engineering, University of Guelph, Guelph, ON N1G 2W1, Canada; bhumi@uoguelph.ca (B.P.); emcbean@uoguelph.ca (E.M.)

**Keywords:** COVID-19, asymptomatic, pre-symptomatic, caseloads, transmission-risk

## Abstract

The numbers of novel coronavirus cases continue to grow at an unprecedented rate across the world. Attempts to control the growth of the virus using masks and social-distancing, and, recently, double-masking as well, continue to be difficult to maintain, in part due to the extent of asymptomatic cases. Analyses of large datasets consisting of 219,075 individual cases in Ontario, indicated that asymptomatic and pre-symptomatic cases are substantial in number. Large numbers of cases in children aged 0–9 were asymptomatic or had only one symptom (35.0% and 31.4% of total cases, respectively) and resulted in fever as the most common symptom (30.6% of total cases). COVID-19 cases in children were more likely to be milder symptomatic with cough not seen as frequently as in adults aged over 40, and past research has shown children to be index cases in familial clusters. These findings highlight the importance of targeting asymptomatic and mild infections in the continuing effort to control the spread of COVID-19. The Pearson correlation coefficient between test positivity rates and asymptomatic rates of −0.729 indicates that estimates of the asymptomatic rates should be obtained when the test positivity rates are lowest as the best approach.

## 1. Introduction

The severe acute respiratory syndrome coronavirus 2 (SARS-CoV-2) that caused the COVID-19 pandemic announced by the World Health Organization in March 2020 has brought about enormous challenges of illness and death and stagnating economies around the world. In attempts to control virus transmissibility, lockdowns of economies and extensive restrictions have been implemented with elaborate quarantine plans, all intended to slow down the potential for the third wave. Even in the US, with the highest detected positive rates and death rates in the world, the percentage of the total population who have tested positive is only approximately 10% of the population, meaning that many new COVID-positive cases will arise and for some, they will be asymptomatic, and hence may be candidates able to continue to spread the COVID-19 virus [1].

Nevertheless, the hidden aspects influencing the assessment and quarantining efforts include the people who are asymptomatic, pre-symptomatic or have mild symptoms, which can translate into people who are not being detected/identified to be tested for positivity for SARS-CoV-2. Lacking a rapid test protocol, cases may not be identified. This continues to be a problem, resulting in increasing importance to gain as much understanding as feasible in the context of asymptomatic infections.

At the time of preparing this paper, the COVID-19 pandemic has caused 118,129,308 cases and 2,621,986 deaths globally [2]. Noteworthy, showing the importance of the “unknown” carriers of the virus, is a study quantifying asymptomatic infection in New York which reported 50% of infections were either asymptomatic or pre-symptomatic [3]. The best estimate for the proportion of asymptomatic infections as provided by Centers for Disease Control and Prevention (CDC) is 40% [4]. A large cohort consisting of 44,000 people with COVID-19 was included in a study conducted in China, where all deaths occurred among critically ill patients with a case fatality ratio of 2.3%. The illness severity was lower for children; 94% of affected children were reported as either asymptomatic or only suffered a mild disease [5]. The age-groups that patients were classified into were: <10, 10–19, 20–29, 30–79 and ≥80 years. Similar to the estimate provided by CDC, recent documented data suggest approximately 40–45% of SARS-CoV-2 infections were asymptomatic persons [6,7,8]. Many individuals who are asymptomatic end up being instead pre-symptomatic, which occurs when symptoms start showing a few days after initially being tested; however, these pre-symptomatic individuals are just as able to infect others as symptomatic individuals [9]. With growing concern to control the spread of the virus amidst the vaccine immunization plans entering the second wave of COVID-19 in Ontario, improved strategies are needed to screen individuals with mild symptoms effectively.

This study used data from the Ministry of Health (MOH) in Ontario and reviews findings from 219,075 individuals who tested positive for COVID-19 in Ontario, Canada, providing guidelines as to magnitudes and the degree to which different age groups experience different symptoms. Further, indicators are provided to interpret how asymptomatic cases may be characterized based on available data as of this time.

## 2. Materials and Methods

The dataset used in this study was obtained from MOH and consisted of 219,075 individual cases that tested positive from over 8.4 million COVID-19 tests performed throughout Ontario between 24 January 2020 and 10 January 2021. The individuals tested consisted of those who requested a test or were required to be tested (e.g., by some type of agency or school).

The positive tested individuals were classified in terms of symptoms in the dataset as cough, fatigue, fever, headache, shortness of breath, sore throat or, lastly, with no symptoms, as asymptomatic. Other variables such as population demographics, co-morbidities and the likely point of acquisition of the virus were also included, associated with each positive tested person. The computer programming language R was used to develop data visualizations from the MOH dataset [10]. In addition, Microsoft Excel (2019) was used to calculated percentages and create tables [11].

## 3. Literature Review

There is a broad range of severity of illnesses from COVID-19, thus asymptomatic infections are important because the virus can still be transmitted to others causing critical illness in the elderly and immunodeficient populations [12]. Han et al. conducted a study of 91 children with a confirmed positive COVID-19 test (between 18 February and 31 March 2020) aged 19 or under in Korea, reporting that 22% were asymptomatic during the monitored period [13]. All patients in the study conducted by Han et al. were put in isolation regardless of whether the infection was asymptomatic, mild or severe, to fully study the spectrum of infection in children. The authors estimated from the data that 85 children (93% of the total children) who tested positive would have been missed if the testing strategy focused on symptomatic children alone [13]. This is worrisome as undocumented COVID-19 cases in children could cause the rapid spread of silent COVID-19 transmission in communities (although given the strict quarantine strategies adopted in Korea, the transmission potential of children was not likely large) [14].

Unfortunately, screening tests that rely upon current lists of symptoms are not very robust at identifying who has the virus due to the number of people who are asymptomatic and/or pre-symptomatic. Hence, the ability to rapidly determine via a test would be enormously valuable. Portable rapid tests are being used in sports (such as curling, rugby and basketball) across Canada where everyone is tested for COVID-19; thus, when implemented in a sector of the population, if all individuals are tested, it is possible to detect asymptomatic people [15]. However, that approach is not yet feasible on the scale of the Province of Ontario.

Since asymptomatic infections are more common in children than in adults, schools and day-cares need to be vigilant to ensure safety [16]. Children in preschool and kindergarten to the 12th grade have a higher number of contacts relative to the rest of the population, with contacts at home, school or leisure, and thus a higher chance to incur physical contacts compared to the workplace or while travelling [17]. Regardless, children pose a high risk of viral circulation within their family and their community, although, for example, a study by Laxminarayan et al. conducted in two Indian states found transmission risk was highest between children of the same age-group with 26% of the contacts testing positive, as opposed to between children and young adults where 5–7% of all contacts tested positive [18]. In the absence of large-scale testing or the ability to aggressively practice contact-tracing, asymptomatic people will not be isolated and, hence, spread the virus. Infectivity studies have confirmed the possibility of transmission during an asymptomatic period [19,20]. Since schools reopened in Ontario, 11 January 2021 for schools in northern Ontario and 25 January 2021 for most schools in southern Ontario, and with the emergence of COVID-19 variants, the socializing among children is of substantial concern for spreading the COVID-19 virus. These findings suggest the importance of re-evaluating the safety in schools and day-care.

Studies investigated by both Zhang et al. and Zhen-Dong et al. described children as the index cases (first confirmed positive case for COVID-19) in familial clusters indicating it would be precarious to neglect the role of children in transmission of COVID-19 [21,22]. Some examples of the age of child index cases noted in these studies include a 3-month-old and a 10-year-old. Thus, this warrants concern for parents deciding whether to send their children back to school. Parents, caregivers, guardians and schoolteachers have to make difficult choices to weigh the risks and benefits of different educational options; should they utilize virtual or in-person learning. Some children may be asymptomatic regardless, however, due to it not being recognized, they go to school and risk transmitting to others.

## 4. Results

Individual positive test cases were categorized as displayed in Table 1 where the data were grouped to estimate the percentages of cases with no symptoms, as well as 1, 2 or ≥3 symptoms. Some individual case data had symptoms labeled as “unknown”; therefore, to ensure accurate analyses, individuals with unknown symptoms were excluded, resulting in 48,328 individual case data, originally from 219,075 individual cases in the MOH dataset. In addition, 95% confidence intervals are calculated and depicted in Table 1 based off their percentile and total number of cases per age group with known symptoms [23].

Apparent from the data summarized in Table 1, the youngest age categories (0–9) and oldest age categories (80–89 and ≥90) have high asymptomatic rates, greater than 30%. All other age categories were 16% or less.

From the six symptoms available in the dataset (cough, fatigue, fever, headache, shortness of breath and sore throat), Table 2 highlights the number of cases observed by symptom and the associated percentage of total cases with ± representing 95% confidence intervals. Cough was the most recorded symptom (25.9% of total cases across all age groups, as seen in Table 2). When each age group was analyzed separately, cough was found to be the first “most common symptom” for ages ≥40, second most common for ages ≥20, but ranked third for children aged 0–9. The most common symptom in children aged 0–9 was “fever” with 30.6% of cases.

**Figure 1:**
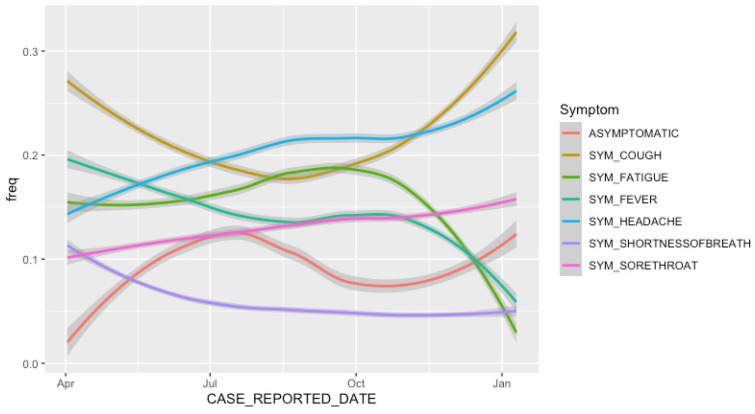
Frequency of symptoms (cough, headache, fatigue, fever, sore throat and shortness of breath) and asymptomatic cases.

To identify how the symptoms being reported have changed over time, Figure 1 plots the frequency of the symptoms highlighted in Table 2 against the case reported date (the date when local public health units were first notified of the positive case). The trend lines in Figure 1 are estimated using a local polynomial regression fit, and the dark grey bands represent 95% confidence intervals. The methodology used to perform polynomial regression was described by Cleveland et al. [24].

Minor symptoms which rank lower in Table 2, such as sore throat, have an increased frequency later on in the pandemic (Figure 1). This suggests Ontario has improved its ability to identify and track less severe symptoms. The asymptomatic line graph in Figure 1, with an increase in the months of Summer 2020 and a decrease from approximately July 2020 to October 2020, is likely a function of the number of tests completed; testing numbers in Ontario were ramped up in July, slowed down during October, and ramped back up again in December 2020.

**Figure 2:**
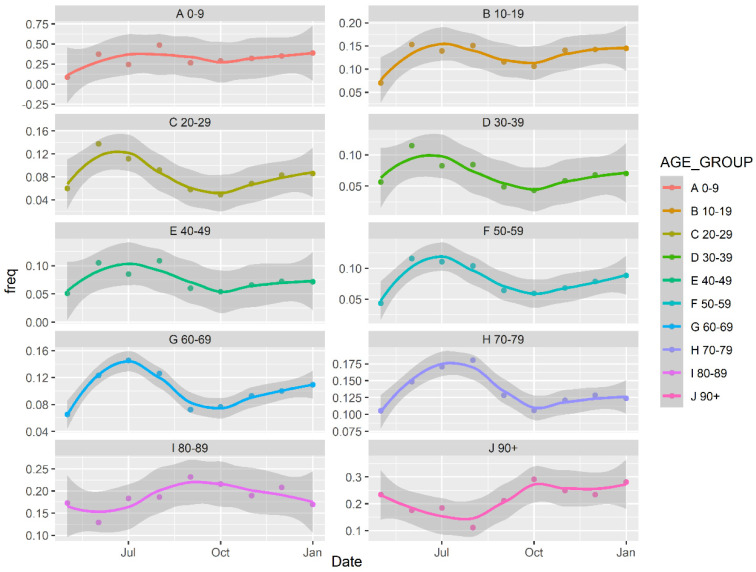
Frequency of asymptomatic cases per age group.

The frequency of asymptomatic cases can be an important determinant if a large number of cases are being identified or missed. Hence, Figure 2 highlights the frequency of asymptomatic cases (i.e., number of asymptomatic cases/total number of cases) for each age group in a monthly scale to better understand the changes as proportions of asymptomatic cases in each age group. Here, again, the trend lines are estimated using local polynomial regression using the methodology described by Cleveland et al. [24].

First, the frequencies of asymptomatic cases were low across all age groups in April 2020. This is likely due to insufficient testing completed at the outset of the pandemic, thus missing less severe/asymptomatic cases. By July 2020, there was a peak in asymptomatic frequency which corresponds to the timing of increased numbers of COVID-19 tests throughout the months of Summer 2020 [25]. It is likely that, during the summer, the largest portion of COVID-19 cases were being identified and thus the high percentage of asymptomatic cases identified. However, for the age groups ranging 0–79, the asymptomatic case frequency decreased from July 2020 to October 2020 and then increased slowly with a linear slope, which indicates many asymptomatic cases were likely not being detected during this time.

To provide further evidence that the asymptomatic rates being detected in Ontario are highly impacted by COVID-19 case detection rates, we compared test positivity rate (a proxy for case detection rates since case detection rates are not available for Ontario) with total asymptomatic rate per month between April 2020 to January 2021. The Pearson correlation coefficient between test positivity rate (a well-established indicator for case detection rate [26]) and asymptomatic rate was calculated to be −0.729. This is a very strong correlation, which suggests that asymptomatic rates are correlated to case detection rates (as case detection improves, and test positivity decreases asymptomatic rates will likely increase). Therefore, the low asymptomatic rates observed in October 2020 for many age categories are more likely a result of insufficient testing levels rather than a change in symptoms, and thus the high asymptomatic rates identified during the summer months, when test positivity rates were lowest, more closely resembles the true asymptomatic rates of COVID-19 patients in Ontario. It is noted, however, that asymptomatic rates for children aged 0–9, do not fluctuate substantially throughout the pandemic, which suggests children asymptomatic rates are less impacted by test positivity.

## 5. Discussion

A notable feature of SARS-CoV-2 is asymptomatic infection, where a fraction of the population infected with the virus do not experience, develop and report symptoms [27]. In Table 1, children in the age group of 0–9 have the largest percentage of asymptomatic infections (35.0% of total cases) and a single symptom (31.4% of total cases). From the reported data, children are more likely to experience mild cases or asymptomatic, presumably, also, least able to articulate the symptoms and therefore greater chances to go undetected. In addition, Leung [28] reported that milder symptoms are often observed in children compared to adults. In children aged 0–9, 30.6% of cases had fever; thus, even a mild fever that resolves quickly should be taken seriously given almost one-third (31.4% with a single symptom and 35.0% asymptomatic) of children experience little to no symptoms. A recent news article indicated that doctors in Toronto warn parents that children with even a single symptom which is mild and resolves quickly should be tested for COVID-19 and stay-at-home awaiting the results, as a crucial procedure to stop viral transmission [29].

Du et al. [30] analyzed and compared epidemiological, clinical, laboratory and radiological characteristics of children (age category 0–16) and adult COVID-19 cases from two research centers in China, early during the course of the pandemic (23 January 2020–15 February 2020). Similar to the findings in this study that note fever was the most common symptom in children aged 0–9, Du et al. found most cases to be mild in children, where fever (35.7%) and dry cough (21.4%) were clinical manifestations in children [30]. Similarly, in a study conducted in China with 134 COVID-19 cases in children, the most common symptom was fever (76.1%) followed by cough [31]. However, Du et al. reported that dry cough and phlegm were more common symptoms in adults compared to children, which was also seen from the findings in this study as cough was the most recorded symptom for ages ≥40 [30]. In addition, the age of asymptomatic children was likely to be younger than that of symptomatic children [30]. Even among asymptomatic children, more than half of the cases had lung injury; thus, the authors concluded that, despite most cases where children were asymptomatic or mild with mild clinical signs and symptoms, there is substantial lung injury, although less clinical disease [30].

Oran and Topol performed a meta-analysis using studies either cross-sectional or longitudinal in design to estimate the proportion of asymptomatic individuals in England and found at least one-third of SARS-CoV-2 infections were asymptomatic, however they did not observe significant differences in the frequency of asymptomatic infections between age groups [32]. Additionally, they found 47% of those individuals who tested positive were asymptomatic (at the time of testing) using data from the large study with random PCR testing of 932,072 participants. From these findings, the indications are that more testing needs to be done, as Ontario may not be identifying a large proportion of positive tests because the identified asymptomatic rates were characterized as much lower (12.1% in December 2020) than in June last year (18.3% in June 2020), both of which were much lower than the rate from UK’s random PCR testing.

## 6. Conclusions

COVID-19 has caused tremendous hardships and challenges around the globe. As cases due to the new COVID-19 variants of concern continue to rise, it is extremely important to detect asymptomatic infections, stay alert and tighten restrictions to help keep the third wave at bay [33]. “Cough” was not a common symptom in younger age groups and asymptomatic people do not cough or sneeze, but these individuals go about their daily routine moving around from one place to another, and hence are able to disseminate the virus to other people. Thus, wearing masks, physical distancing and other such non-pharmaceutical interventions protect everyone from asymptomatic cases; not only protecting the wearer from becoming a positive case, but also, since many people are asymptomatic or pre-symptomatic, this protects others from the wearer if the wearer may be asymptomatic/pre-symptomatic. Given the high number of asymptomatic cases in children (35.0%), it is very important that everyone understands they are helping others by adopting good practices (wearing a mask and keeping social distancing).

## Figures and Tables

**Table 1:** Proportion of total cases with 0, 1, 2, or ≥3 symptoms.

	% of Total Cases				Total
Age Group	0 Symptoms	1 Symptom	2 Symptoms	≥3 Symptoms	Number of Cases with Known Symptoms
0–9	35.0 ± 2.7	31.4 ± 2.6	22.3 ± 2.3	11.3 ± 1.8	1216
10–19	11.4 ± 0.9	28.0 ± 1.2	29.5 ± 1.2	31.1 ±1.2	5276
20–29	6.6 ± 0.4	22.4 ± 0.7	27.9 ± 0.8	43.0 ± 0.9	12,458
30–39	6.8 ± 0.5	21.3 ± 0.9	27.3 ± 0.9	44.6 ± 1.1	8562
40–49	7.6 ± 0.6	21.8 ± 0.9	27.2 ± 1.0	43.5 ± 1.1	7341
50–59	7.8 ± 0.6	20.8 ± 1.0	27.7 ± 1.1	43.7 ± 1.2	6829
60–69	8.8 ± 0.9	21.6 ± 1.3	27.8 ± 1.4	41.8 ± 1.6	3818
70–79	15.2 ± 1.8	24.2 ± 2.2	24.9 ± 2.2	35.6 ± 2.4	1522
80–89	31.2 ± 3.1	20.1 ± 2.7	25.8 ± 2.9	22.9 ± 2.8	877
≥90	42.2 ± 4.7	18.9 ± 3.7	24.0 ± 4.0	14.9 ± 3.4	429

**Table 2:** Number of cases and percent of total cases with symptoms of cough, headache, fatigue, fever, sore throat and shortness of breath.

Rank	Symptom	Number of Cases	%
1	Cough	56785	25.9 ± 0.4
2	Headache	50132	15.8 ± 0.3
3	Fatigue	34643	15.1 ± 0.4
4	Fever	33155	22.9 ± 0.5
5	Sore throat	32127	6.4 ± 0.3
6	Shortness of breath	14049	14.7 ± 0.6

## Data Availability

The data are not publicly available to protect privacy. Data are available from the Public Health Case and Contact Management Solution (CCM), extracted by Public Health Ontario (contact via https://www.publichealthontario.ca/en/data-and-analysis/using-data/data-requests) (accessed on 3 February 2021) for researchers who meet the criteria for access to confidential data.
